# Molecular subtypes and scoring tools related to Foxo signaling pathway for assessing hepatocellular carcinoma prognosis and treatment responsiveness

**DOI:** 10.3389/fphar.2023.1213506

**Published:** 2023-08-24

**Authors:** Sheng Tu, Yunqing Qiu

**Affiliations:** State Key Laboratory for Diagnosis and Treatment of Infectious Diseases, National Clinical Research Center for Infectious Diseases, Collaborative Innovation Center for Diagnosis and Treatment of Infectious Diseases, The First Affiliated Hospital, College of Medicine, Zhejiang University, Hangzhou, China

**Keywords:** hepatocellular carcinoma, Foxo signaling pathway, molecular subtypes, prognosis, treatment assessment

## Abstract

**Background:** Transcription factors in Foxo signaling pathway influence hepatocellular carcinoma metastasis through epithelial mesenchymal transition-related pathways. Prognostic factors in the Foxo signaling pathway are feasible for HCC prognosis and therapeutic management.

**Methods:** Based on the differentially expressed genes and Foxo signaling pathway genes in HCC, the ConsensusClusterPlus package was conducted to identify Foxo signaling pathway-related molecular subtypes in HCC. Based on the DEGs in the FMSs, the optimal prognostic factors in HCC were screened by cox and least absolute shrinkage and selection operator (LASSO) cox analysis to form the Foxo prognosis score (FPS). The prognostic predictive effectiveness of FPS was assessed by Kaplan Meier (K-M) analysis and Receiver Operating Characteristic (ROC) analysis. Additionally, tumor microenvironment (TME) score, tumor mutation burden (TMB) and treatment sensitivity differences in FMSs and FPS groups were also evaluated.

**Results:** There were low, medium and high Foxo signaling pathway activity molecular subtypes in HCC named FMS 1, FMS 2 and FMS 3, respectively. FMS 1 with lowest Foxo signaling pathway activity presented an excellent survival advantage, while FMS 3 with highest Foxo signaling pathway activity exhibited an inhibitory TME status. According to FPS grouping, low FPS exhibited favorable survival, low TMB and anti-tumor activity. Patients in the low FPS group were mostly in the early stage of cancer. Moreover, we found that patients with high and low FPS exhibited different sensitivity to chemotherapy, and patients with low FPS were more sensitive to immunotherapy.

**Conclusion:** We revealed a novel molecular subtype and prognostic tool based on Foxo signaling pathway signature, which could potentially provide a direction for accurate and effective assessment of potential personalized treatment options and prognostic management for HCC patients.

## Introduction

Hepatocellular carcinoma (HCC) is the most prevalent pathological type of liver cancer, with 90% of liver cancer cases being HCC (Llovet et al., 2021). According to the prognostic model of cancer pathology by the World Health Organization (WHO), more than 1 million HCC deaths were expected (Llovet et al., 2021). In current clinical practices, liver resection, transplantation, and chemotherapy remained the dominant options for the treatment of HCC. Surgical resection demonstrated good five-year survival in the treatment of HCC, with five-year survival rates of approximately 70%–80%, however, there were postoperative liver dysfunction and recurrence difficulties, maintaining liver function in HCC patients after surgery was reported to be the key challenge (European Association for the Study of the Liver, 2018). Liver transplantation was accepted as the superior HCC treatment option to surgical resection in clinical practice, with more than half of HCC liver transplant patients achieving postoperative survival of 10 years or more, however, the waiting time for donor in liver transplantation was an uncertainty (Llovet et al., 2021). In contrast, chemotherapy was predominantly compromised by tumor heterogeneity, with different individuals exhibiting diverse treatment progression (Vogel et al., 2022). Therefore, postoperative prognostic management of HCC was the substantial challenge in clinical practice, and accurate prognostic assessment tools were integral to improve the survival rate of HCC.

The transcription factors in the Foxo signaling pathway, FOXO1 and FOXO3, contributed critical proteins to the development of malignant progression of HCC (Yang et al., 2021). In HCC, Akt signaling normally occurs in an active state, with overactive AKT signaling inhibiting FOXO1 transcriptional processes. Regular FOXO1 transcription inhibited epithelial mesenchymal transition (EMT) and transforming growth factor-β (TGF-β) expression, whereas dysregulation of EMT and TGF-β expression promoted HCC cell invasion and metastasis to other tissues when the FOXO1 transcription process was blocked (Dong et al., 2017). FOXO3 exhibited normal expression levels in normal liver tissues but was abnormally highly expressed in liver tissues of HCC patients, with further studies also confirming that FOXO3 contributed to the suboptimal disease-free survival and prognosis of HCC (Ahn et al., 2018; Song et al., 2020).

In this study, we integrated high-throughput sequencing data of HCC from multiple databases (The Cancer Genome Atlas, GENE EXPRESSION OMNIBUS) to conduct a comprehensive theoretical analysis. The intention of this study was aimed to establish molecular subtypes of Foxo signaling pathway activity in HCC, followed by an attempt to construct the prognostic scoring system for HCC based on the specialized genes in the subtypes. To our knowledge, this is the first time that Foxo signaling pathway was studied in the prognosis of liver cancer.

## Materials and methods

### Data collection

RNA-seq data for the TCGA-LIHC sequencing project were sourced from the Cancer Genome Atlas (TCGA, https://portal.gdc.cancer.gov/) database. This study was conducted using the HCC samples cohort in TCGA as the training set. To validate the results of this investigation, three additional datasets were accessed as validation sets. RNA-Seq data for HCC sequencing researches were accessed from HCCDB (Lian et al., 2018) (http://lifeome.net/database/hccdb/home.html) (project name: ICGC-LIRI-JP) and GENE EXPRESSION OMNIBUS (GEO) database (https://www.ncbi.nlm.nih.gov/gds) (registration number: GSE14520 (Li et al., 2022), GSE76427 (Grinchuk et al., 2018)), respectively. Additionally, clinical information of samples in TCGA-LIHC, ICGC-LIRI-JP, GSE14520 and GSE76427 were also collected. The genes in the Foxo signaling pathway were sourced from the Kyoto encyclopedia of genes and genomes (KEGG) database (https://www.genome.jp/kegg/) (Supplementary Table S1).

### Data pre-processing

Clinical data and RNA-Seq data of samples in TCGA-LIHC, ICGC-LIRI-JP, GSE14520 and GSE76427 were imported into the sangerbox database (Shen et al., 2022) for the following processing. Samples with missing clinical data were excluded; samples with survival time >0 were retained; ensemb information was converted to Gene symbol; gene with multiple probe information were averaged as the expression data of the gene. After processing, 355 HCC samples and 50 control samples were included in TCGA-LIHC; 212 HCC samples were maintained in ICGC-LIRI-JP; 221 and 115 HCC samples were maintained in GSE14520, GSE76427. Detailed information was listed in Table 1.

**TABLE 1:** Clinical information of HCC samples in TCGA-LIHC, ICGC-LIRI-JP, GSE14520 and GSE76427.

Characteristics	TCGA-LIHC (*N* = 355)	ICGC-LIRI-JP (*N* = 212)	GSE76427 (*N* = 115)	GSE14520 (*N* = 221)
Status				
Alive	226	176	92	136
Dead	129	36	23	85
Age				
Mean ± SD	59.81 ± 13.10			
Median [min-max]	61.00[16.00,90.00]			
Gender				
FEMALE	115			
MALE	240			
T.stage				
T1	175			
T2	87			
T3	77			
T4	13			
Unknow	3			
N.stage				
N0	241			
N1	3			
Unknow	111			
M.stage				
M0	256			
M1	3			
Unknow	96			
Stage				
I	166			
II	80			
III	82			
IV	3			
Unknow	24			
Grade				
G1	53			
G2	169			
G3	117			
G4	11			
Unknow	5			

For the data analyzed in this study please see: https://www.jianguoyun.com/p/DcQQQIoQ7NfMCxiwxYUFIAA.

### Identification of Foxo Molecular subtypes

In TCGA-LIHC, the limma package (Ritchie et al., 2015) was performed to screen differentially expressed genes (DEGs) in HCC samples based on the expression matrix of HCC samples and control samples (|log2FC|>1 & FDR<0.05). The expression matrix of genes in the Foxo signaling pathway was extracted and the univariate COX model was performed to identify HCC prognosis-related genes (*p* < 0.05). Overlapping genes in prognosis-related genes in DEGs and Foxo signaling pathway were extracted, and the consistency clustering analysis was performed based on the expression matrix of overlapping genes. The ConsensusClusterPlus R package (Wilkerson and Hayes, 2010) was performed to execute the consistency clustering analysis. The clustering parameters were set as follows: metric distance: km algorithm and euclidean; number of bootstraps: 500; number of clusters range: *k* = 2–10. Foxo Molecular subtypes (FMS) in HCC were determined according to the consistency matrix and consistency cumulative distribution function for each k-value range.

### Protein-protein interaction network analysis

The limma package was used to perform differential analysis to identify DEGs in each FMS according to the gene expression matrix in the different FMSs. The DEGs were imported into the STRING database (Szklarczyk et al., 2021) to construct the Protein-protein interaction network (PPI network) with parameters set to: confidence score > 0.7. The PPI network was imported into Cytoscape software (version: 3.9.1) (Shannon et al., 2003) for MCODE sub-network clustering and topology analysis. Genes in the sub-networks with the highest scores based on MCODE algorithm were included for subsequent analysis.

### Construction of Foxo prognosis score

Based on the gene expression matrix in MCODE 1, the coxph function in the survival package (Therneau and Lumley, 2015) was performed for univariate COX model analysis to initially screen for HCC prognosis-related genes. The Least absolute shrinkage and selection operator COX model was performed to reduce the model fit, and the penalty parameter lambda was selected by 10-fold cross-validation method. The model under the best lambda value was selected for multivariate COX model construction, and the step AIC function in the MASS package was performed for optimal model selection, and the model under the minimum value of Akaike information criterion (AIC) was considered the optimal model. The Foxo prognosis score (FPS) was constructed based on the gene regression coefficients (β) and expression data in the model. The evaluation equation was:

$$FPS=\sum \beta_{i}*{expression}_{i}$$

### Prognostic guidance value of FPS

The FPS of samples in TCGA-LIHC was calculated according to the FPS evaluation equation. Samples with FPS>0 was defined as high FPS group and samples with FPS<0 was defined as low FPS group. The survival package was performed to perform Kaplan-Meier (K-M) survival analysis and graphed K-M survival curves. The timeROC package (Blanche et al., 2013) was performed for Receiver Operating Characteristic (ROC) analysis and ROC curves were graphed. The prognostic guidance value of FPS was validated in the external validation cohorts, CGC-LIRI-JP, GSE14520 and GSE76427.

### Mutational landscape analysis of genes in the Foxo signaling pathway

The somatic mutation data corresponding to HCC samples in TCGA-LIHC were also sourced from the TCGA database. The Maftools package (version: 2.8.05) (Mayakonda et al., 2018) was deployed to perform copy number variation (CNV) and Single nucleotide variant (SNV) analysis. The tmb function in the Maftools package was performed to assess the level of Tumor mutation burden (TMB) in HCC. Based on Thorsson et al. (2018), molecular mutation characteristics of TCGA-LIHC were captured to assess the Aneuploidy Score, Fraction Altered, Number of Segments, and Number of Segments levels in HCC samples.

### Scoring or abundance analysis of infiltrating immune cells in the immune microenvironment

For HCC samples in TCGA-LIHC, multiple tumor microenvironment (TME) infiltration algorithms were conducted to assess the immune score or infiltration abundance of infiltrating immune cells in these samples. The CIBERSORT algorithm (Chen et al., 2018) was developed to assess the infiltrative abundance of 22 immune cells in HCC samples; the MCP-Count (Becht et al., 2016), and TIMER algorithms (Li et al., 2020) were developed to assess the immune scores of immune cells in TME. The GSVA package (Hanzelmann et al., 2013) was performed to perform single-sample gene set enrichment analysis based on the 28 immune cell gene sets in the study by Barbie et al. (2009).

### Pathway analysis

The h.all.v7.5.1.symbols.gmt gene set from MSigDB database was imported from the GSEA website for single sample gene set enrichment analysis (ssGSEA) via the GSVA package. The Hmisc package (Harrell and Harrell, 2019) was employed for spearman correlation analysis between the pathway ssGSEA score and FPS.

### Evaluation of immunotherapy/chemotherapy sensitivity

The present study also sought to reveal the sensitivity of HCC patients in different FMS and FPS groups to immunotherapy as well as chemotherapeutic drug treatment. Immune checkpoint gene expression levels in HCC samples were assessed according to the immune checkpoint gene in the study of Auslander et al. (2018). Next, the Tumor Immune Dysfunction and Exclusion (TIDE) score, interferon gamma (IFNG), exclusion score, dysfunction score, and myeloid-derived suppressor cells (MDSCs) score of HCC samples were accessed in the TIDE (http://tide.dfci.harvard.edu/login/) (Jiang et al., 2018) website. Chemotherapeutic drugs, Erlotinib, Saracatinib, TGX221, Roscovitine, GNF-2, CGP-082996, Pyrimethamine, NSC-87877, treated HCC Sequencing data were sourced from the Genomics of Drug Sensitivity in Cancer (GDSC, https://www.cancerrxgene.org/) database. pRRophetic package (Geeleher et al., 2014) was developed to assess the half maximal inhibitory concentration (IC50) values of these drug-treated HCC data.

### Assessment of the clinical value of FPS and construction of nomogram

Age, Gender, Stage, Grade, and FPS information of HCC samples in TCGA-LIHC were extracted and univariate COX and multivariate COX analyses were performed to identify pivotal independent prognostic parameters in HCC for Nomogram construction. Meanwhile, the calibration curves were performed to assess the prognostic predictive value of Nomogram based on the 1-year, 3-year, and 5-year survival rates of HCC predicted by Nomogram versus the actual survival rates of recorded HCC. Additionally, Decision curve (DCA) was graphed for assessing the clinical predictive value of FPS, Nomogram based on its information.

### Statistical analysis

All statistical analyses were performed using R software (version 4.0.3). Moreover, Sangerbox (http://sangerbox.com/home.html) also assisted in data processing. *p* value < 0.05 was treated as statistically significant.

## Results

### Genomic alterations of prognosis-related Foxo signaling pathway genes in HCC

**FIGURE 1:**
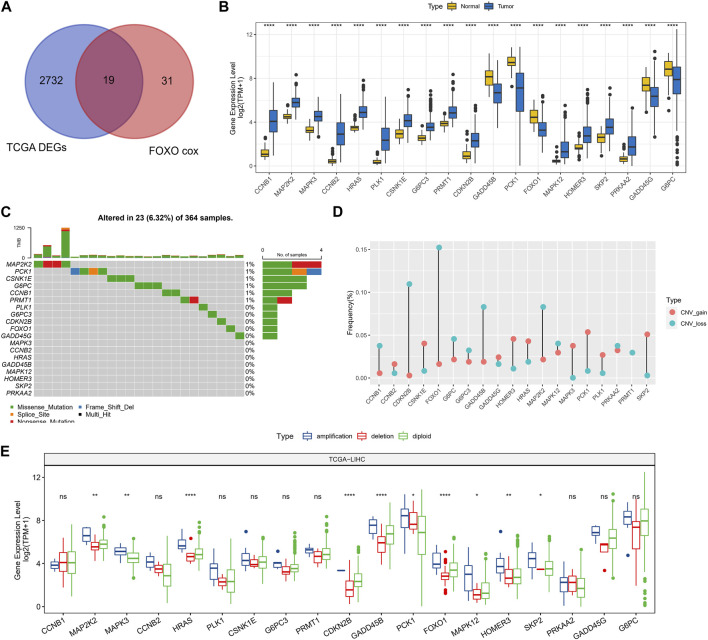
Prognosis-related genomic alterations of Foxo signaling pathway genes in HCC samples. **(A)** Wayne diagram showing overlapping genes of prognosis-related Foxo signaling pathway genes and DEGs in HCC. **(B)** 19 - Gene expression in tumor tissue and normal tissue. **(C)** Mutational landscape of 19 genes in tumor samples. **(D)** 19-gene CNV landscape in HCC samples. **(E)** 19-Gene expression levels in the CNV amplification group, CNV deletion group, and CNV diploid group. *p < 0.05, **p < 0.01, ****p < 0.0001, ns, no difference.

In TCGA-LIHC, 2751 DEGs were screened out between HCC and normal tissues and prognosis-related Foxo signaling pathway genes were screened, with 19 genes co-existing in them (Figure 1A). Among these genes, 5 genes expressed highly in control tissues and 14 genes expressed highly in tumor tissues (wilcox.test, *p* < 0.05) (Figure 1B). Subsequently, the mutation status of 19 genes in HCC tissues was analyzed with 23 HCC samples (6.32%) in which these genes were mutated. Missense Mutation and Nonsense_Mutation constituted the most frequent type of mutations (Figure 1C). 19-genes were also estimated for CNV, for which we found genes exhibited lower CNV amplification or deletion (Figure 1D). Finally, according to CNV status, HCC samples were divided into CNV amplification, CNV deletion and CNV diploid groups, and 19-gene expression was evaluated in all three groups. We found that these genes exhibited higher expression levels in the CNV amplification group overall (Figure 1E).

### Foxo Molecular subtypes in HCC

**FIGURE 2:**
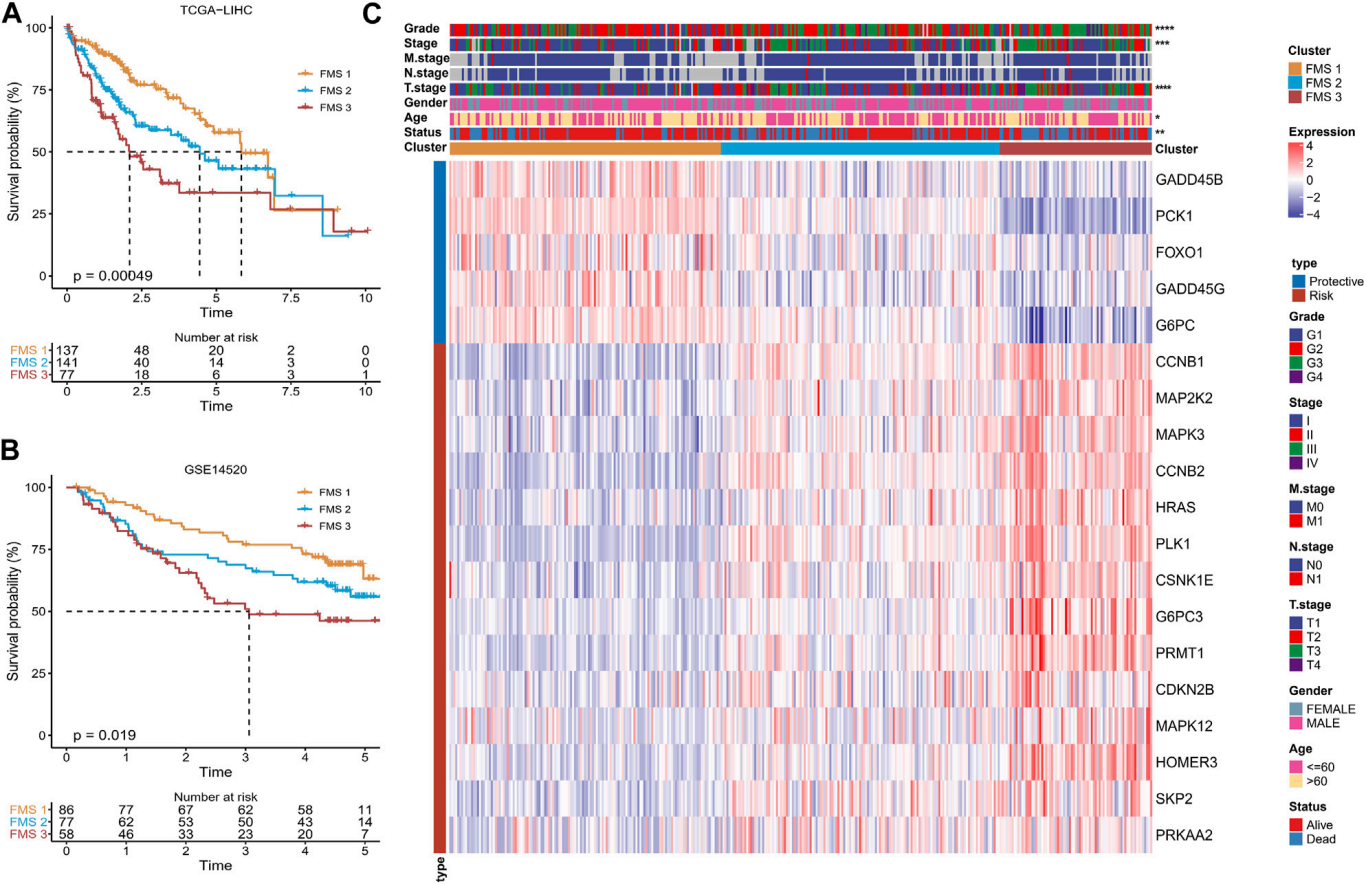
Differences in survival, clinical characteristics among FMSs. **(A–B)** K-M Ssurvival curve of FMSs in TCGA-LIHC, GSE14520. **(C)** Heatmap showing 19-gene expression and clinicopathological information in FMSs. *p < 0.05, **p < 0.01, ***p < 0.001, ****p < 0.0001.

Following the 19-genes expression matrix, HCC samples in TCGA-LIHC were performed consistency clustering analysis to uncover the molecular subtypes concerning Foxo signaling pathway signature (FMS). When *k* = 3, the clustering of HCC patients exhibited excellent consistency with low interference between samples (Supplementary Figure S1A–C). Therefore, we defined 3 molecular subtypes in HCC named FMS 1, FMS 2, and FMS 3. The molecular subtypes were further verified in the samples of GSE14520, and the trends were consistent (Supplementary Figure S1D–F). In TCGA-LIHC and GSE14520, FMS 1 both exhibited an excellent survival benefit (Figures 2A, B). In TCGA-LIHC, from the statistical information of clinical information and 19-gene expression heatmap in FMS 1-3, we could clearly observe that FMS 1 exhibited remarkably high expression of protective factors and remarkably low expression of risk factors. FMS 3 exhibited the contrast tendency. Therefore, FMS 1, FMS 2, and FMS 3 were defined as the low activity cluster, medium activity cluster, and high activity cluster of Foxo signaling pathway, respectively. Moreover, we also noted that patients with HCC in FMS 3 exhibited high clinical stage features (Figure 2C).

### TME differences in FMSs

**FIGURE 3:**
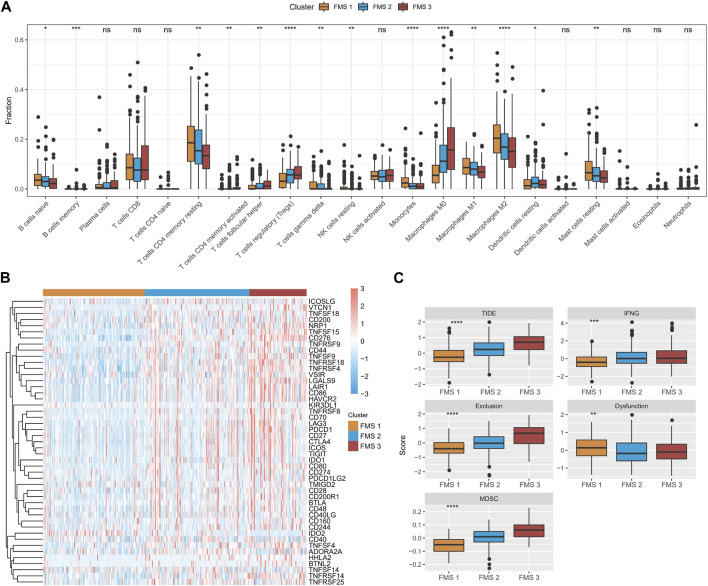
TME differences between FMSs. **(A)** CIBERSORT results in FMSs, demonstrating the abundance of 22 immune cell infiltrates. **(B)** Immune checkpoint gene expression levels in FMSs. **(C)** TIDE score, IFNG score, Exclusion score, Dysfunction score, MDSC score. *p < 0.05, **p < 0.01, ***p < 0.001, ****p < 0.0001, ns, no difference.

According to CIBERSORT results, B cells naïve, T cells CD4 memory resting, Monocytes, Macrophages M1, Mast cells resting expressed high infiltration abundance in FMS 1; T cells regulatory (Tregs), Macrophages M0 expressed high infiltration abundance in FMS 3 (Figure 3A). Depending on the ssGSEA, MCP-Count, and TIMER results, we observed that higher levels of immune cell infiltration scores were exhibited in FMS 3 (Supplementary Figure S2). Additionally, lower levels of immune checkpoint gene expression were measured in FMS 1, and the highest level of immune checkpoint gene expression was demonstrated in FMS 3 (Figure 3B). We further remarked that TIDE score, IFNG score, Exclusion score, MDSC score appeared to be higher in FMS 3 (Figure 3C).

### Protein-protein interaction network in HCC

**FIGURE 4:**
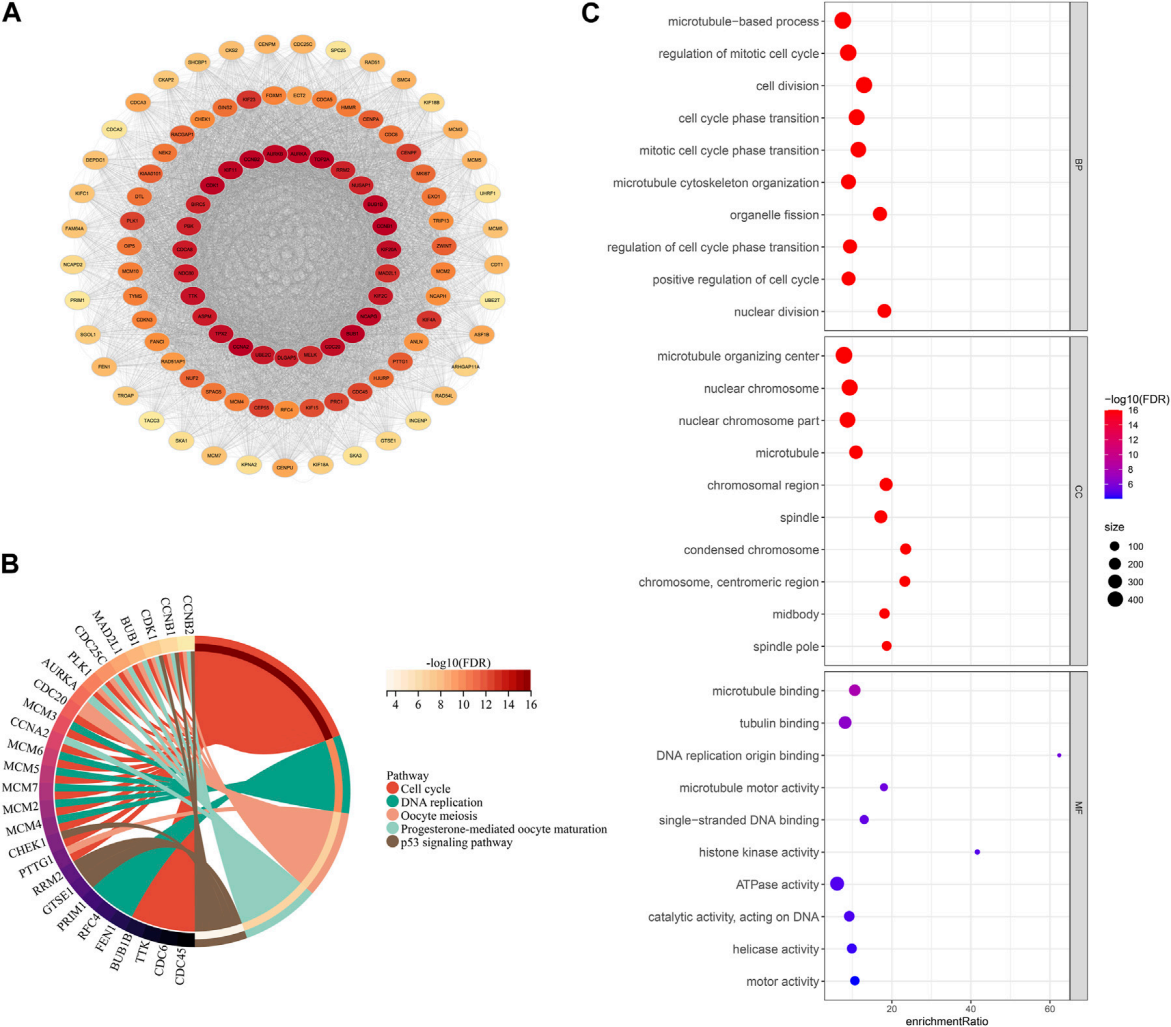
PPI network and functional analysis. **(A)** PPI network for DEGs in FMSs (The darker the color of a single gene, the more it interacts with other genes, indicating its higher importance). **(B)** KEGG chord diagram of genes in MCODE 1. **(C)** GO bubble map of genes in MCODE 1.

To explore the prognostic differences and TME activity differences, differential analysis was performed in FMS 1-3 to construct PPI networks to explore the protein regulatory network differences among FMSs. Firstly, differential analysis was performed. 576 DEGs were presented in FMS 1, consisting of 188 upregulated expression DEGs and 388 downregulated expression DEGs; 2 upregulated expression DEGs were presented in FMS 2; 1,048 DEGs were presented in FMS 3, consisting of 658 upregulated expression DEGs and 390 downregulated expression DEGs. 1,108 overlapping DEGs were presented in FMSs, and these genes were included in STRING to construct PPI network, and 717 genes were included in PPI network with confidence score >0.7 (Figure 4A). According to the MCODE algorithm in cytoscape software, the sub-network MCODE 1 with the highest large MCODE degree value in the PPI network was retained for functional enrichment analysis, containing 103 genes. These genes were remarkably enriched in cell cycle, DNA replication, oocyte meiosis, progesterone-mediated oocyte maturation, and p53 signaling pathway (Figure 4B). The GO results further evidenced that these genes were closely interlinked with DNA replication transcriptional processes, such as, regulation of mitotic cell cycle, cell division, cell cycle phase transition, and mitotic cell cycle phase transition (Figure 4C).

### FPS for assessing HCC prognosis

**FIGURE 5:**
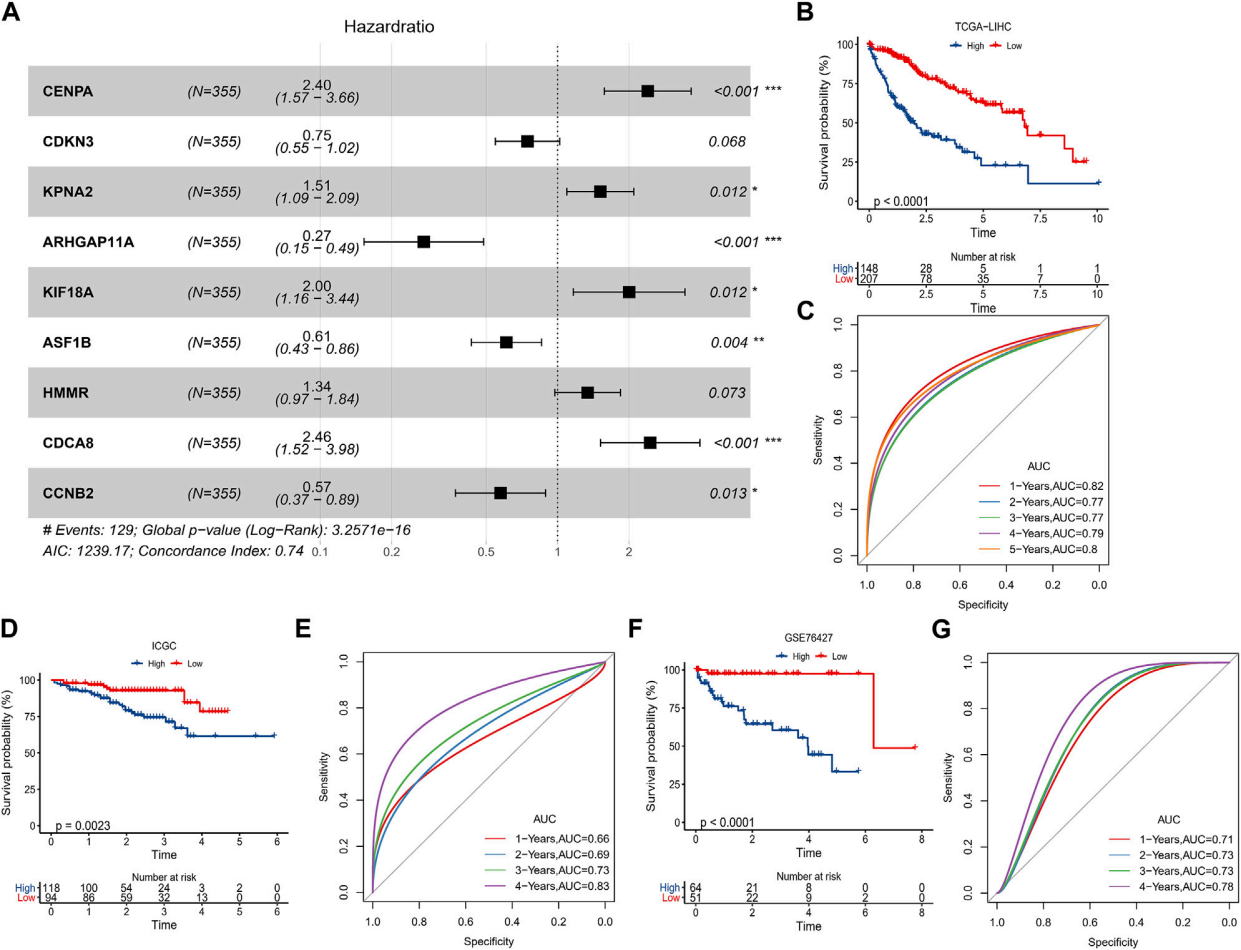
Predictive value of FPS. **(A)** Multivariate COX forest plot of 9-prognostic factors. **(B, C)** K-M survival curve, ROC curve in TCGA-LIHC. **(D, E)** K-M survival curve, ROC curve in ICGC-LIRI-JP. **(F, G)** K-M survival curve, ROC curve in GSE76427. *p < 0.05, **p < 0.01, ***p < 0.001.

In TCGA-LIHC, 92 prognosis-related genes were identified by univariate COX analysis, we found that these genes were all Risk genes. According to the trajectory diagram of the change of independent variable coefficients in LASSO COX analysis, the interference of similar genes in the model was minimized at lambda = 0.0172, and the model fit was minimized at this time, when 16 genes were included in the model (Supplementary Figure S3A, B). Finally, CENPA, CDKN3, KPNA2, ARHGAP11A, KIF18A, ASF1B, HMMR, CDCA8, and CCNB2 were screened out by multivariate COX analysis and were considered to be the optimal model to compose the HCC prognostic scoring system with FPS = 0.875*CENPA+(-0.291*CDKN3)+0.414*KPNA2 +(-1.298*ARHGAP11A)+0.694*KIF18A+(-0.496*ASF1B)+ 0.292*HMMR+0.899*CDCA8+(-0.554*CCNB2) (Figure 5A). The prognostic differences between the high FPS group (FPS>0) and the low FPS group (FPS<0) according to the FPS = 0 grouping and the prognostic accuracy of FPS were assessed by K-M survival curves and ROC curves, respectively. Patients with HCC in the low FPS group presented remarkable survival advantage (Figure 5B). The AUC values of FPS predicting 1-year, 2-year, 3-year, 4-year, and 5-year survival of HCC were 0.82, 0.77, 0.77, 0.79, and 0.8 (Figure 5C). The prognostic value of FPS was further validated in ICGC-LIRI-JP, GSE76427, and GSE14520, we observed that patients with low FPS in all three datasets exhibited remarkable survival advantage with FPS demonstrated higher AUC values (Figures 5D–G, Supplementary Figure C, D).

### Prognostic performance of FPS in pathological subgroups

**FIGURE 6:**
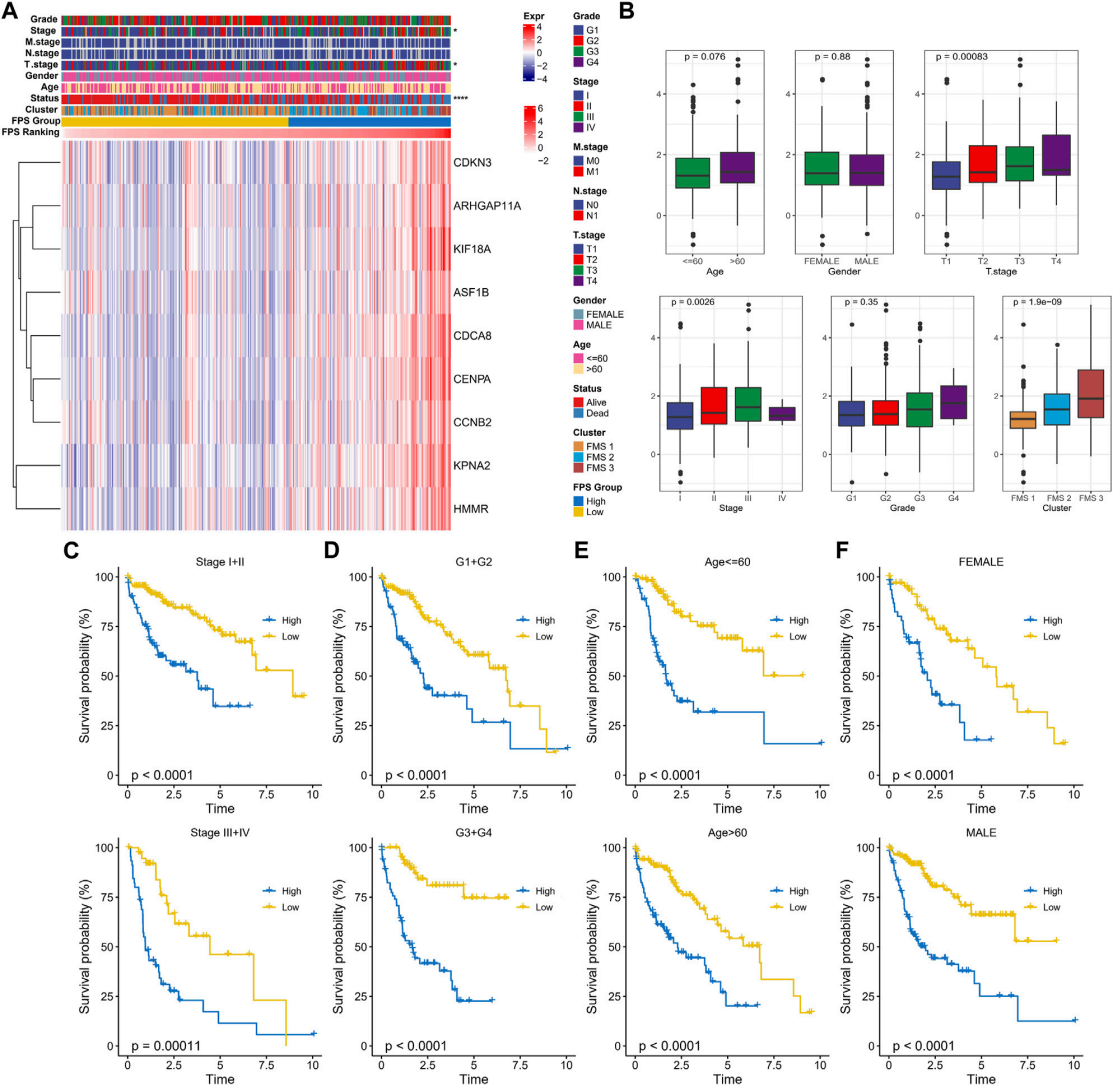
Clinical characteristics in the FPS groups. **(A)** Heat map of 9-prognostic factor expression in FPS groups combined with clinical characteristics of patients. **(B)** FPS differences in clinicopathological groups. **(C–F)** K-M survival curves of patients in high and low FPS groups in Stage I + II, Stage III + IV, G1+G2, G3+G4, Age<=60, Age>60, FEMALE, and MALE groups. *p < 0.05, ****p < 0.0001.

In TCGA-LUAD, the clinicopathological statistical information, 9-gene expression level, and FPS distribution of HCC samples were demonstrated in Figure 6A. We observed that there were remarkable differences in Stage, T stage, and Status of patients in the high FPS group and low FPS group, and the 9-gene expressed higher levels in the high FPS group. In subgroups including Stage, FPS in Age, T stage, Grade, and FMS Clusters showed elevated trends with advanced pathological stage. Also, there is also a slight trendy of differences in FPS between men and women (Figure 6B). In Stage subgroups (Stage I + II, Stage III + IV), Grade subgroups (G1+G2, G3+G4), Age subgroups (Age<=60, Age>60), and Gender subgroups (Female, Male), the low FPS group all exhibited remarkable survival advantage (Figures 6C–F).

### Characterization of pathway and genomic variants in FPS groups

**FIGURE 7:**
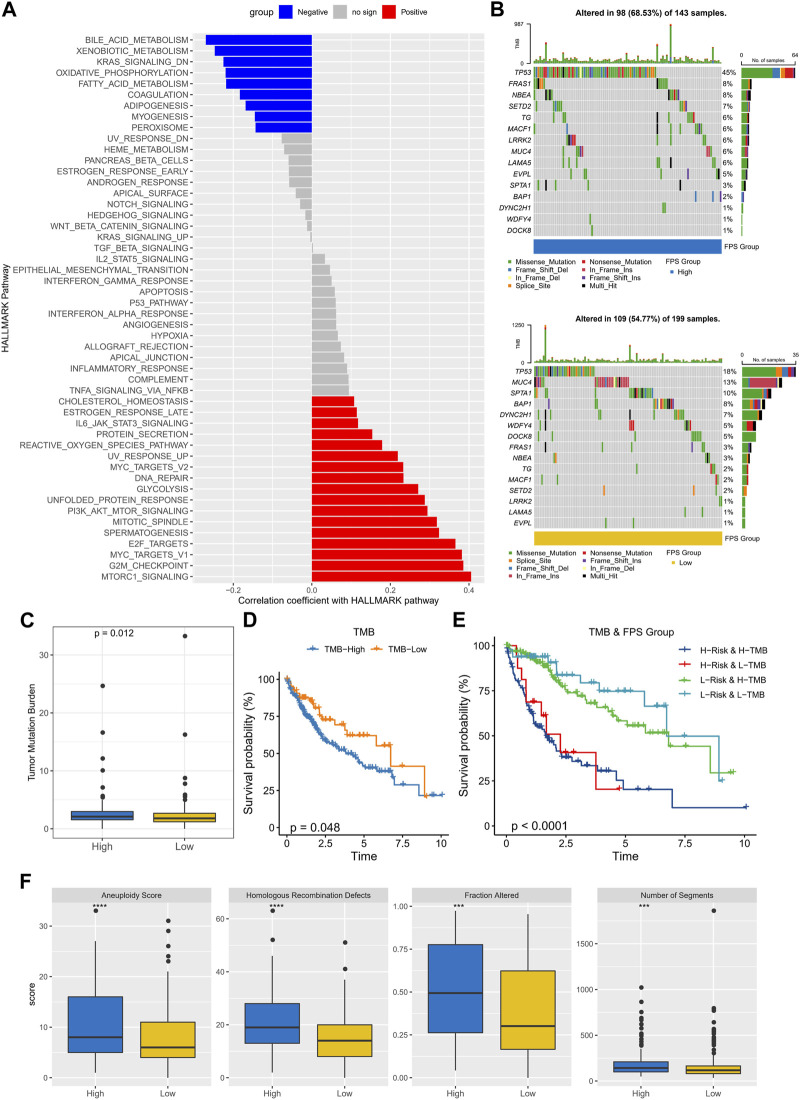
Genomic mutation statistics and pathway differences in FPS groups. **(A)** Biological pathways significantly associated with FPS. **(B)** Mutation landscape in high and low FPS groups. **(C)** TMB in high and low groups. **(D)** K-M survival curves of TMB groups. **(E)** K-M survival curves in patients with TMB combined with FPS groups. **(F)** Homologous Recombination Defects, Aneuploidy Score, Fraction Altered, Number of Segments in high and low groups. ***p < 0.001, ****p < 0.0001.

To observe the connection between FPS and biological functions, the spearman correlation between ssGSEA scores and FPS was evaluated for each pathway in the h.all.v7.5.1.symbols.gmt gene set. We found that FPS exhibited remarkably negative correlations with metabolism-related pathways and remarkably positive correlations with cell cycle-related pathways (Figure 7A). Further, we found differences in gene mutations between high and low FPS groups. The top five mutated genes in the high FPS group were TP53, FRAS1, NBEA, SETD2, and TG; the top five mutated genes in the low FPS group were TP53, MUC4, SPTA1, BAP1, and DYNC2H1. TP53 was more frequently mutated in the high FPS group (Figure 7B). Whereas, the high FPS group exhibited higher TMB (Figure 7C). Patients in the low-TMB group also exhibited remarkable survival advantage (Figure 7D). HCC patients with low TMB and low FPS developed remarkable survival advantage compared with those with high TMB and high FPS scores (Figure 7E). Moreover, we found that the high FPS group exhibited higher Aneuploidy Score, Fraction Altered, Number of Segments, and Number of Segments compared to the low FPS group (Figure 7F).

### Differences in treatment sensitivity and TME in the FPS groups population

**FIGURE 8:**
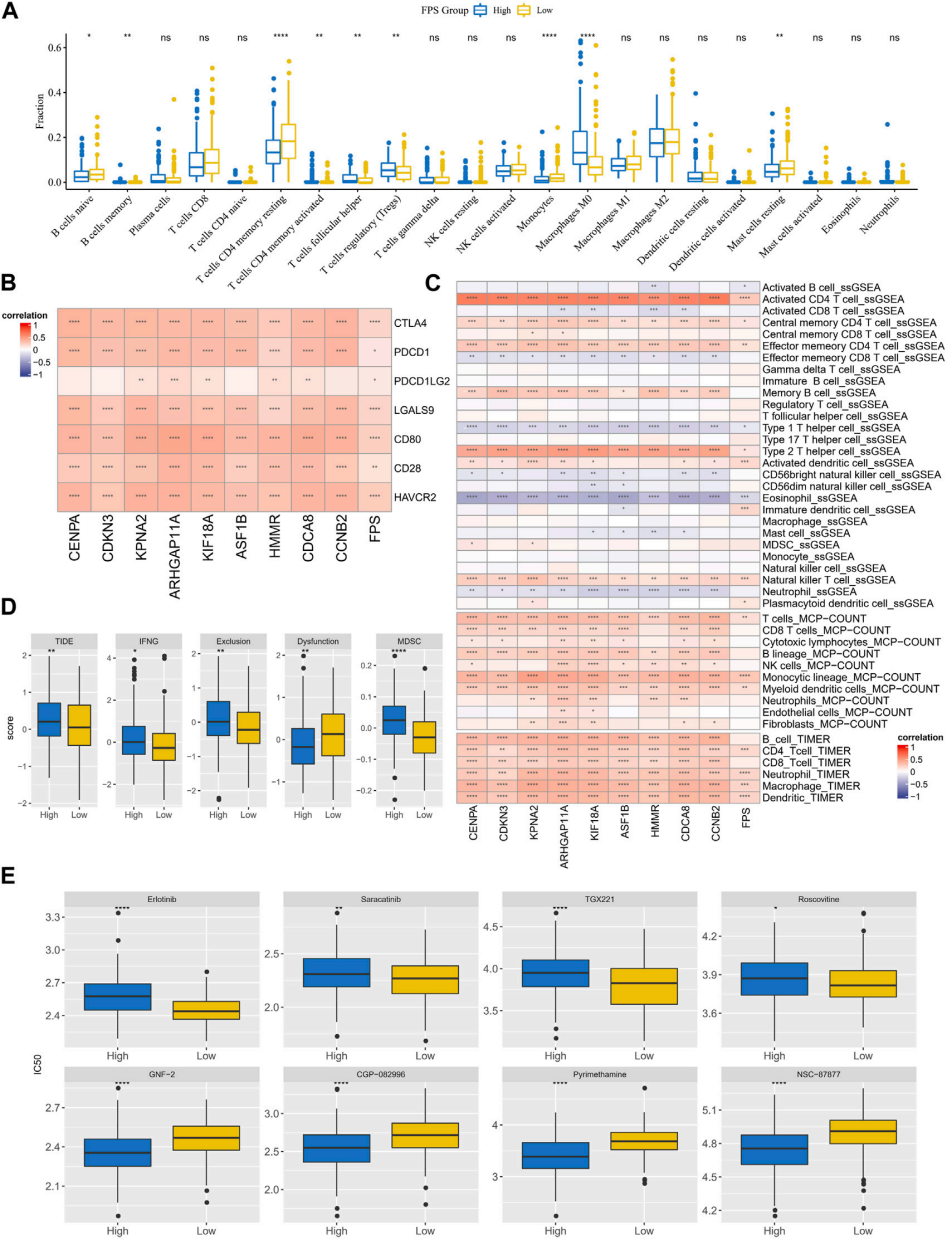
TME differences and treatment sensitivity in FPS groups. **(A)** CIBERSORT results in FPS groups, demonstrating the abundance of 22 immune cell infiltrates. **(B)** Correlation between FPS and immune checkpoint expression, 9-prognostic factor expression. **(C)** Correlation of FPS with ssGSEA, MCP-Count, and TIMER immune scores. **(D)** TIDE score, FNG score, Exclusion score, Dysfunction score, MDSC score in low and high FPS groups. **(E)** IC50 of Erlotinib, Saracatinib, TGX221, Roscovitine, GNF−2, CGP−082996, Pyrimethamine, NSC−87877 in low and high groups. *p < 0.05, **p < 0.01, ***p < 0.001, ****p < 0.0001, ns, no difference.

The immune cell infiltration characteristics in HCC samples in FPS grouping were then characterized. According to the CIBERSORT results, T cells regulatory (Tregs), Macrophages M0 exhibited higher infiltration abundance in the high FPS; B cells naïve, T cells CD4 memory resting, Mast cells resting exhibited higher infiltration abundance in the low FPS group (Figure 8A). FPS exhibited remarkable positive correlation with immune checkpoint gene expression and 9-gene expression levels (Figure 8B). According to the immune cell immune score in ssGSEA, MCP-Count, and TIMER algorithms, FPS exhibited remarkable positive correlation with most immune cell immune scores (Figure 8C). Moreover, we found that the TIDE score, IFNG score, Exclusion score, Dysfunction score, and MDSC score were remarkably higher in the high FPS group than in the low FPS group (Figure 8D). In addition, the sensitivity of patients with different FPS to chemotherapeutic agents according to its characteristics was estimated. Notably, patients in the low FPS group were more sensitive to treatment with Erlotinib, Saracatinib, TGX221, and Roscovitine; patients in the high FPS group were more sensitive to treatment with GNF-2, CGP-082996, Pyrimethamine, NSC -87877 were more sensitive (Figure 8E).

### Nomogram plots predicting HCC survival

**FIGURE 9:**
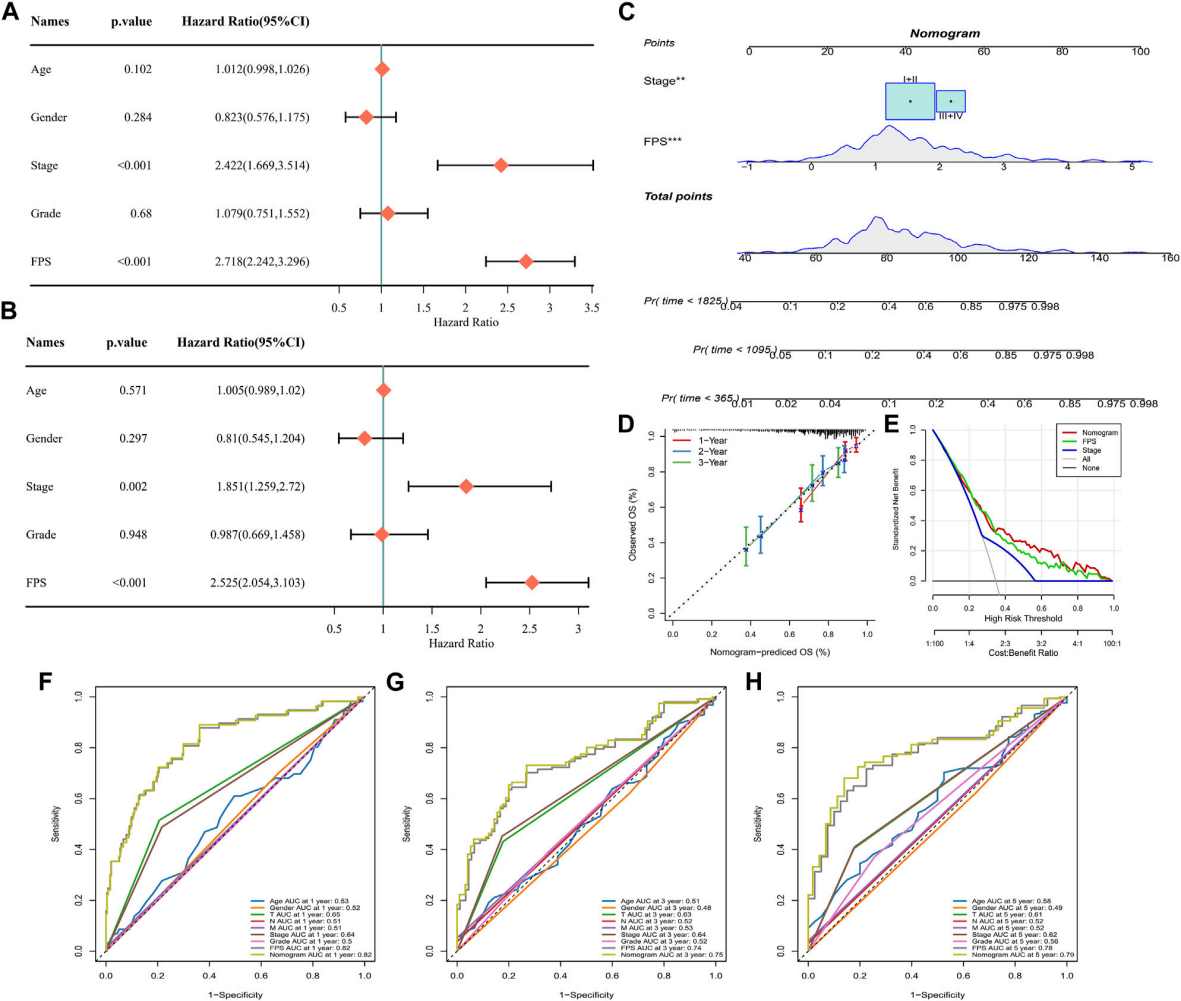
Nomogram for Predicting HCC Survival. **(A–B)** Univariate and multivariate COX analysis of Age, Gender, Stage, Grade, and FPS in TCGA-LIHC. **(C)** Nomogram constructed from Stage and FPS information. **(D)** Calibration curves for 1 year, 3 years, 5 years survival. **(E)** Decision curve. **(F–H)** ROC curves for multiple factors predicting 1-year, 3-year, and 5-year survival in HCC.

Univariate COX and multivariate COX analyses were performed in TCGA-LIHC by integrating information on Age, Gender, Stage, Grade, and FPS. We identified FPS and Stage as independent clinical prognostic factors for HCC (Figures 9A, B). Therefore, with FPS and Stage information, we constructed nomogram for HCC prognosis (Figure 9C), and the calibration curve indicated that Nomogram predicted 1-year, 3-year, and 5-year survival and actual observations of HCC with high fitting tendency (Figure 9D). According to DCA, we observed that nomogram and FPS possessed excellent clinical observation value (Figure 9E). Finally, we integrated information of Age, Gender, TNM Stage, Stage, Grade, FPS, and nomogram to graph ROC curves for predicting 1-year, 3-year, and 5-year survival of HCC. We found that FPS and nomogram possessed the highest AUC values (Figures 9F–H), therefore, FPS and nomogram were trustworthy prognostic scoring tools for HCC.

## Discussion

HCC remains a major cause of cancer death progressing rapidly (Llovet et al., 2021). Inhibitors of key genes in the Foxo signaling pathway were proposed to be an influential factor for novel targeted therapies for HCC (Sun et al., 2021). Therefore, identification of key regulators in the Foxo signaling pathway was essential to optimize HCC survival and treatment options.

Although a lot of progress in systemic therapy, for example, molecular targeting reagents, HCC is one of the worst prognostic tumors attributed to drug tolerance as well as frequent recurrence and metastasis (Oura et al., 2021). As research deepens, TME is receiving an increasing attention, and for this reason, immunosuppressive therapy has been launched (Llovet et al., 2022). A previous study showed that FOXO1 played a part in macrophages through transcriptionally controlling IRF-1/nitrio oxide (NO) axis and reduced the secretion of IL-6 from macrophages in TME indirectly (Cui et al., 2023). In order to Further explore the relationship between Foxo signaling pathway and TME, we also evaluated the immune landscapes in 3 FMSs molecular subtypes. Interestingly, the FMSs exhibited different immune landscapes. Tregs in the TME of FMS 3 patients exhibited increased infiltration abundance. Previous studies indicated that Tregs were significantly enriched and amplified in progressive HCC with the amplified Tregs leading to CD8^+^ T cells being depleted or their function being suppressed (Zheng et al., 2017). Another study revealed that in HCC, Tregs infiltration abundance was reduced in TME by inducing apoptosis to inhibit liver carcinogenesis (Zhou et al., 2021). Additionally, lower levels of immune checkpoint gene expression were discovered in FMS 1, and the highest level of immune checkpoint gene expression was demonstrated in FMS 3. Among these immune checkpoint genes, highly expressed CTLA4, LAG3 and TIGIT has been treated as diagnostic biomarkers for colorectal cancer (Sasidharan Nair et al., 2018). These findings altogether may account for the reason for excellent survival advantage in FMS 1 and provide promising therapy targets toward immune checkpoint gene expression discrepancy for HCC patients. The discovery of FMSs would contribute to refine HCC classification and further shed insights into the connections between Foxo signaling pathway, TME and targeted therapies for immune checkpoint gene.

FPS, a prognostic scoring system for HCC, was constrained based on the DEGs in FMSs. HCC patients with high TMB typically exhibited suboptimal prognostic status (Owada-Ozaki et al., 2018). The combined survival analysis of FPS and TMB showed that patients with low FPS and low TMB exhibited an excellent prognostic status. Lower TIDE score, IFNG score, Exclusion score, Dysfunction score, MDSC score were realized in low FPS, suggesting that low FPS are more sensitive to immunotherapy (Peng et al., 2021). In addition, Nomogram, constructed in accordance with FPS, was a potential tool for clinicians to accurately prognosticate patients based on their FPS characteristics.

9 key prognostic genes were screened out to construct an FPS model. Several of them were related to FOXO pathways. Numerous studies have shown that SIRT1, a member of the silent information regulator 2 (Sir2) family, plays an important role in the deacetylation of FOXO and regulation of autophagy in cells (Singh and Ubaid, 2020). Based on FOXO pathways genes, CENP-A was selected. A literature reported that SIRT7 could promote CENP-A assembly in nucleosome and restrain the tumorigenesis in gut (Liu et al., 2020). SIRT7 is also a member of the Sir2 family. Our findings may indicate that the Sir2 gene family has complex regulatory effects on FOXO pathways. Cyclin B2 (CCNB2) belongs to Foxo signaling pathway and is regarded as a promising biomarker for prognosis in LIHC (Li et al., 2021). Anti-silencing function 1b (ASF1b) was discovered as an oncogenic indicator for gastric cancer (Chen et al., 2022). Other study uncovered that ASF1B activated PI3K/AKT/mTOR signaling to promote cisplatin resistance in triple-negative breast cancer cells (Wang et al., 2022). Intestinally, GSEA analysis revealed that cell division cycle associated 8 (CDCA8) regulated gene sets relevant to PI3K/AKT/mTOR signaling (Bi et al., 2018). Collectively, we excavated some genes which were directly or indirectly related to Foxo signaling pathway or Foxo signaling pathway activity.

Notwithstanding original molecular subtype and prognostic tool based on Foxo signaling pathway signature was developed for HCC patients, some limitations need to be solved for future applying. First of all, more clinical statistics are necessary to calibrate the model. Secondly, 9 gens selected for FPS riskscore signature requires more studies on mechanism to confirm their roles as biomarkers for HCC prognosis.

## Conclusion

Overall, we defined novel molecular subtypes in HCC in accordance with genes in the Foxo signaling pathway, providing new research perspectives in the study of HCC tumor heterogeneity. In this study, we also constructed the HCC prognostic scoring system for Foxo signaling pathway activity characteristics based on DEGs in FMSs, which demonstrated excellent robustness in assessing HCC prognosis, immune activity, and mutational status. Encouragingly, the FPS also exhibited excellent and promising outcome in guiding HCC drug selection. The clinical value of FPS was not further validated in a large sample of clinical cases. Further prospective trials are needed to investigate the prognostic value and therapeutic guidance of FPS in the future.

## Data Availability

The original contributions presented in the study are included in the article/Supplementary Material, further inquiries can be directed to the corresponding author.

## Abbreviations

HCC, Hepatocellular carcinoma; WHO, World Health Organization; EMT, epithelial mesenchymal transition; TGF-β, transforming growth factor-β; TCGA, The Cancer Genome Atlas; GEO; Gene expression omnibus; KEGG, Kyoto encyclopedia of genes and genomes; DEGs, differentially expressed genes; FMS, Foxo Molecular subtypes; AIC, Akaike information criterion; FPS, Foxo prognosis score; K-M, Kaplan-Meier; ROC, Receiver Operating Characteristic; CNV, copy number variation; SNV, Single nucleotide variant; TMB, Tumor mutation burden; TME, tumor microenvironment; ssGSEA, single sample gene set enrichment analysis; TIDE, Tumor Immune Dysfunction and Exclusion; IFNG, interferon gamma; MDSCs, myeloid-derived suppressor cells; GDSC, Genomics of Drug Sensitivity in Cancer; IC50, half maximal inhibitory concentration; DCA, Decision curve; Tregs, T cells regulatory.
