# Supplementary figures and images for: Molecular subtypes and scoring tools related to Foxo signaling pathway for assessing hepatocellular carcinoma prognosis and treatment responsiveness
Source: Front Pharmacol. 2023 Aug 24;14:1213506. doi: 10.3389/fphar.2023.1213506 (PMC10483071; doi:10.3389/fphar.2023.1213506)

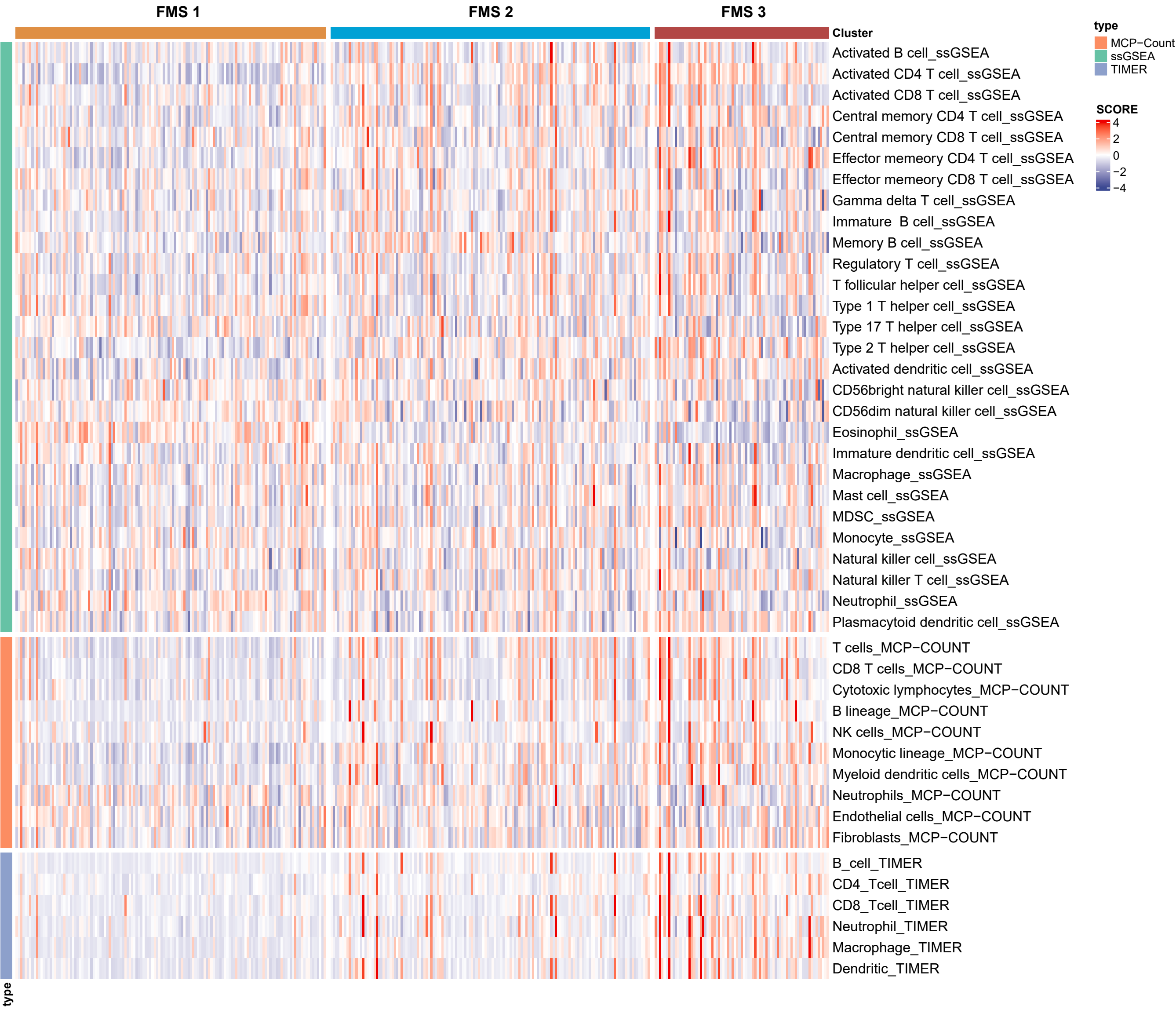

Supplement: Supplementary file 1 [file DataSheet2.PDF]

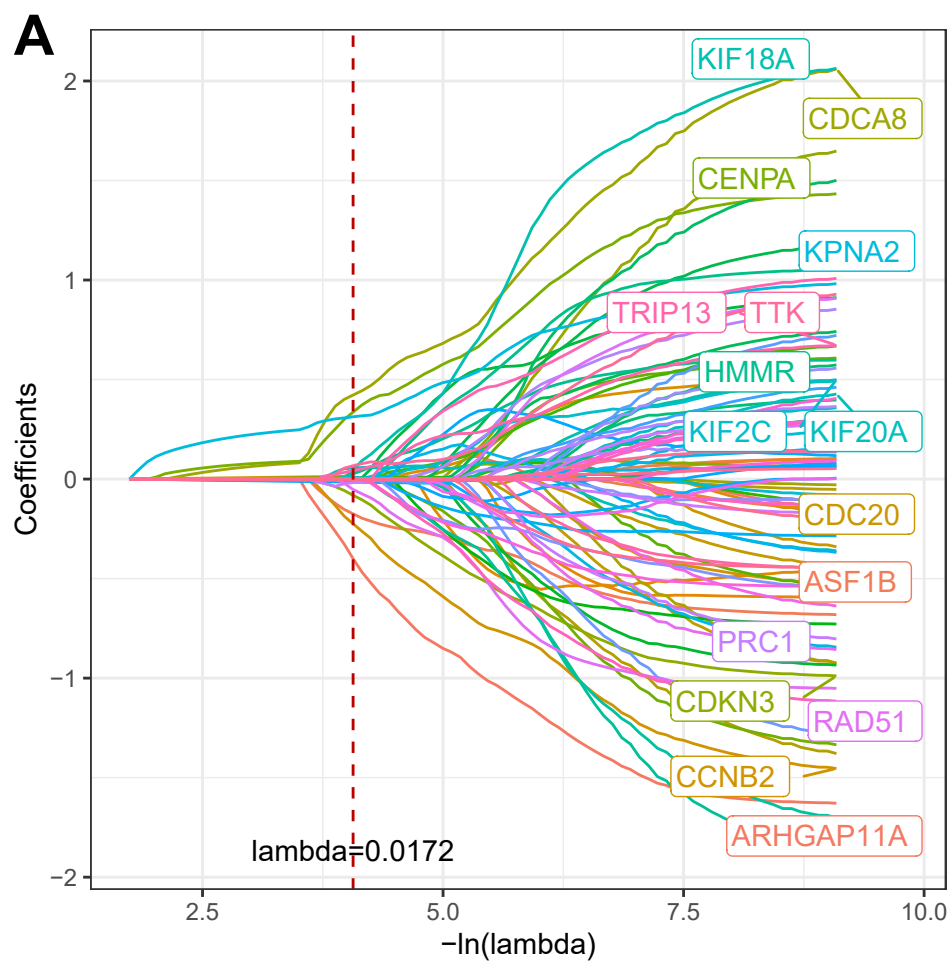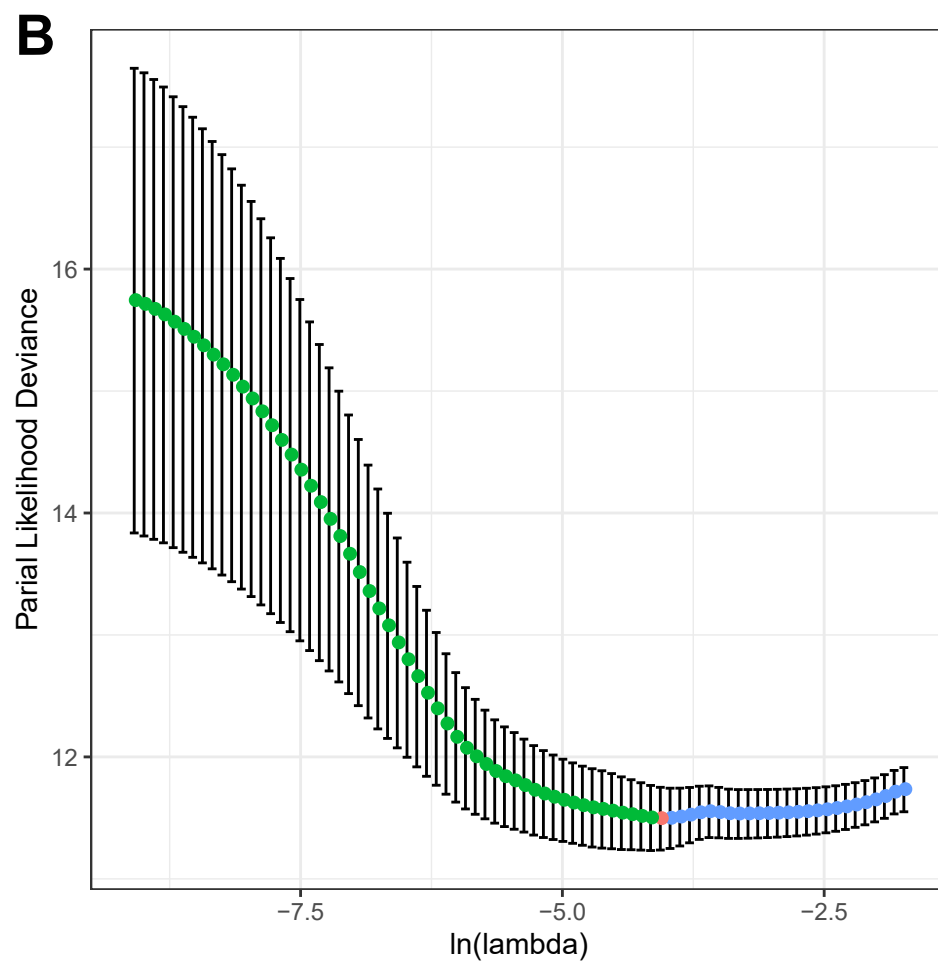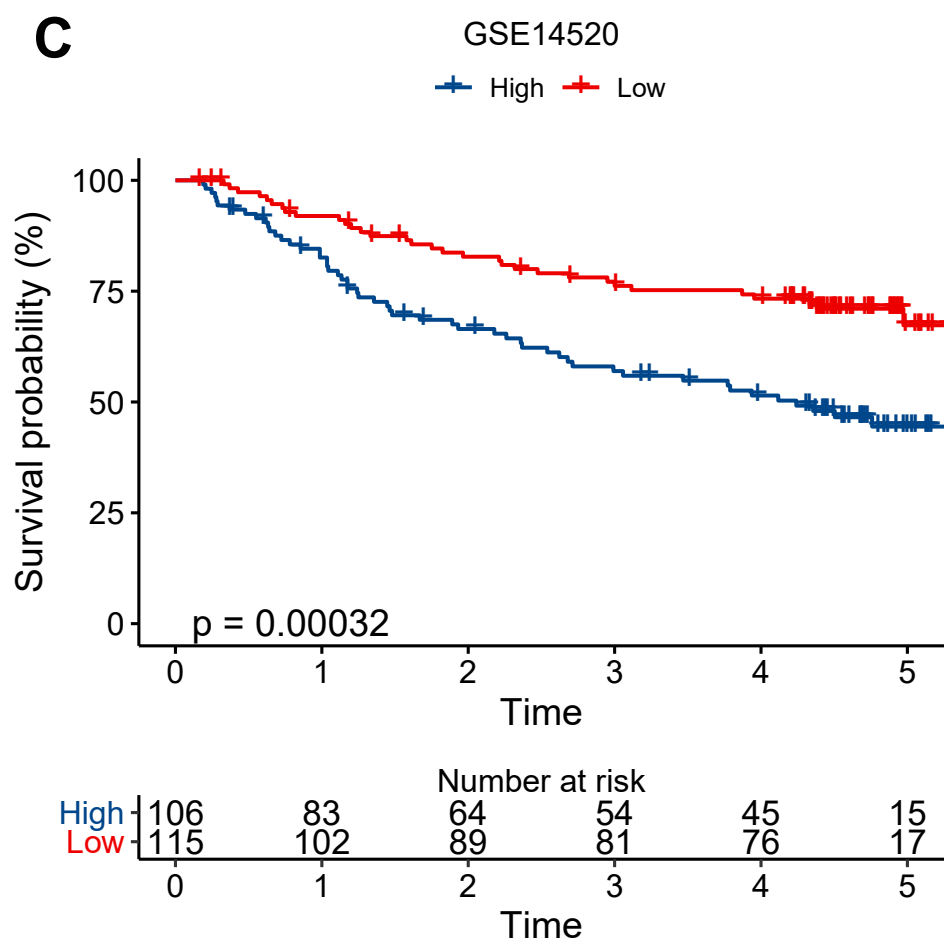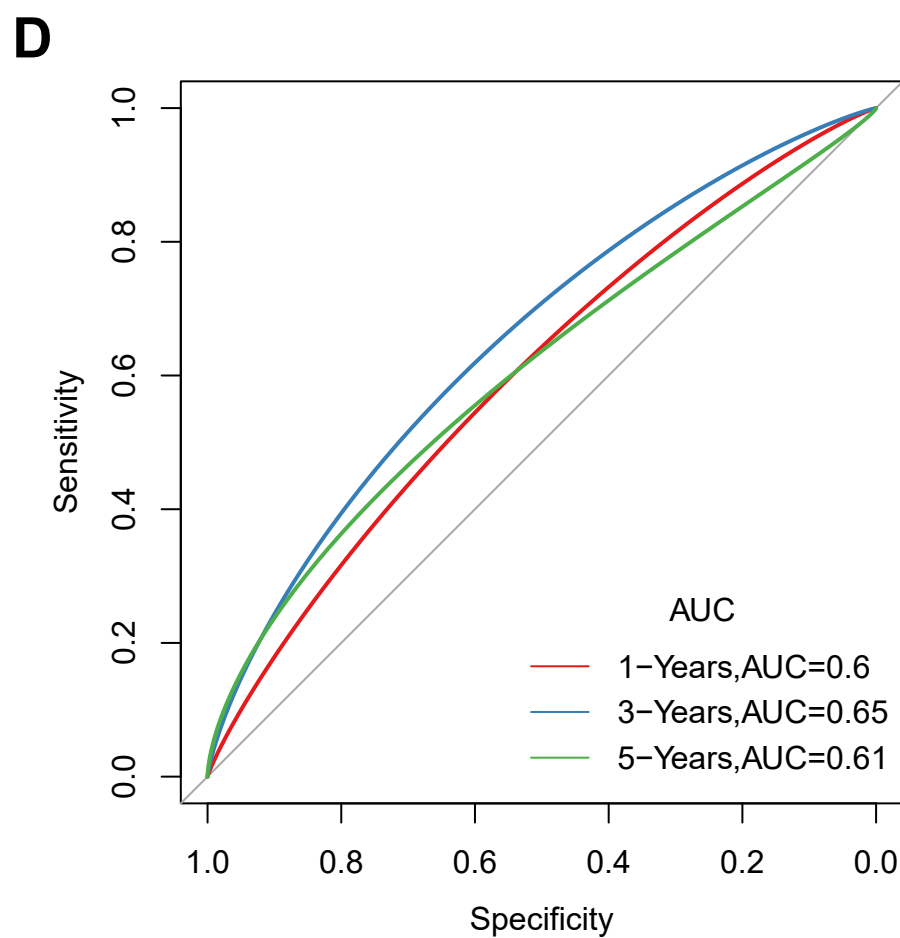

Supplement: Supplementary file 2 [file DataSheet3.PDF]

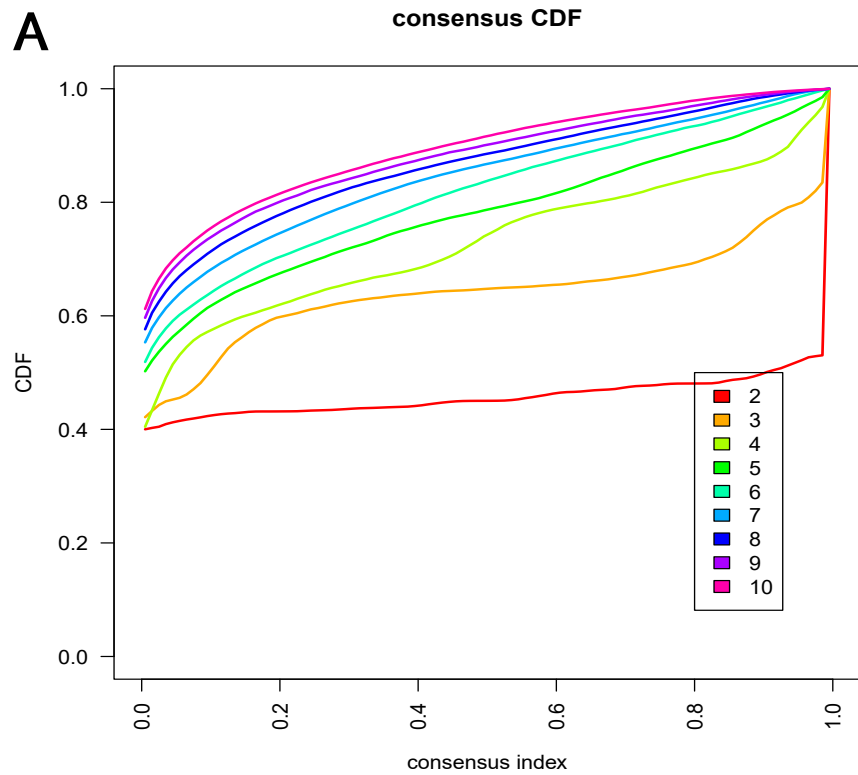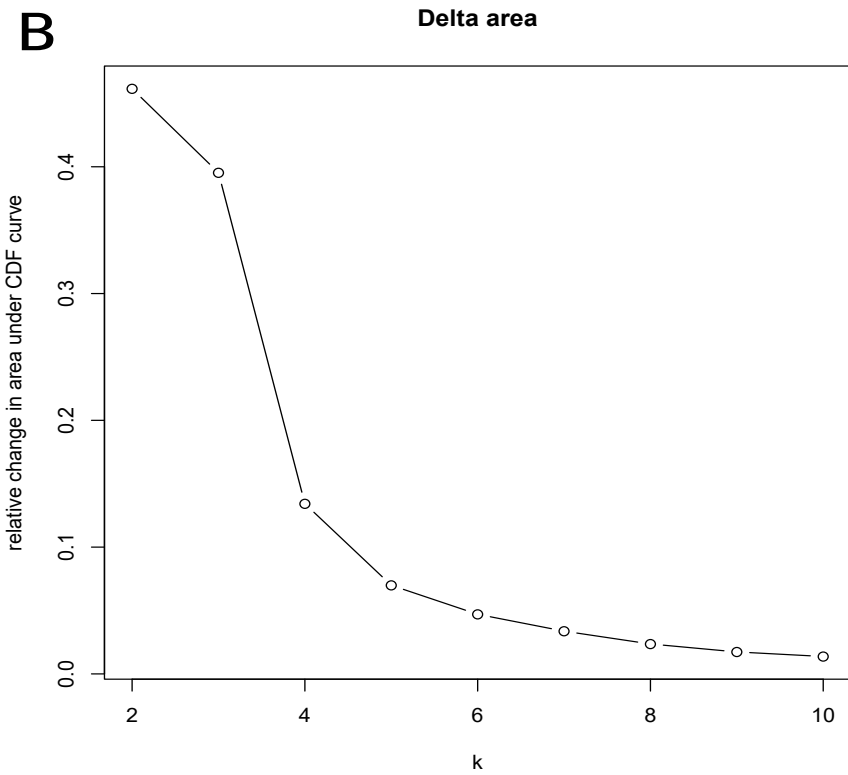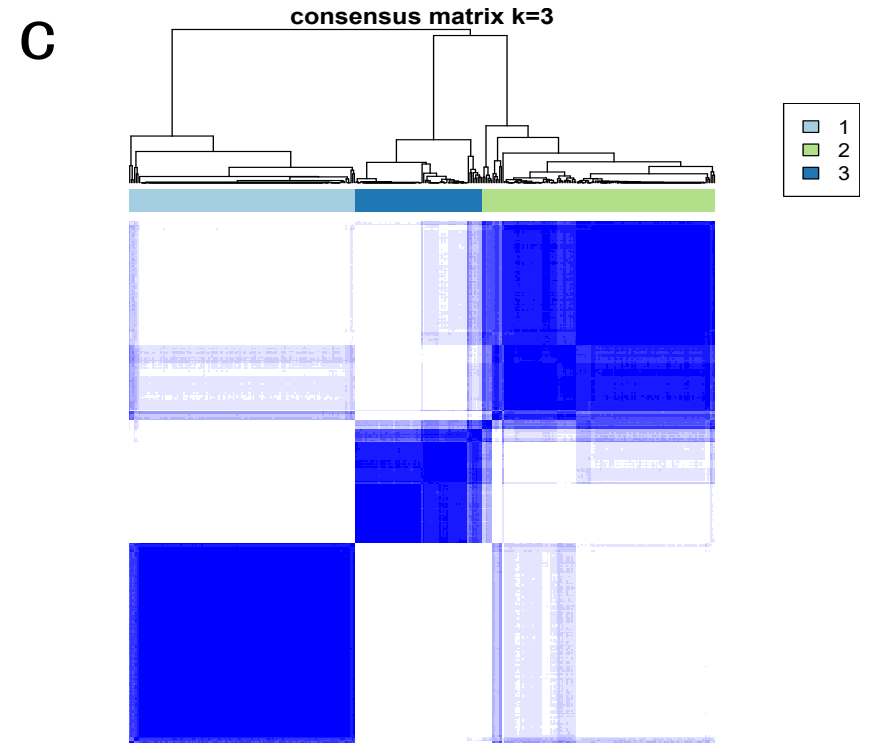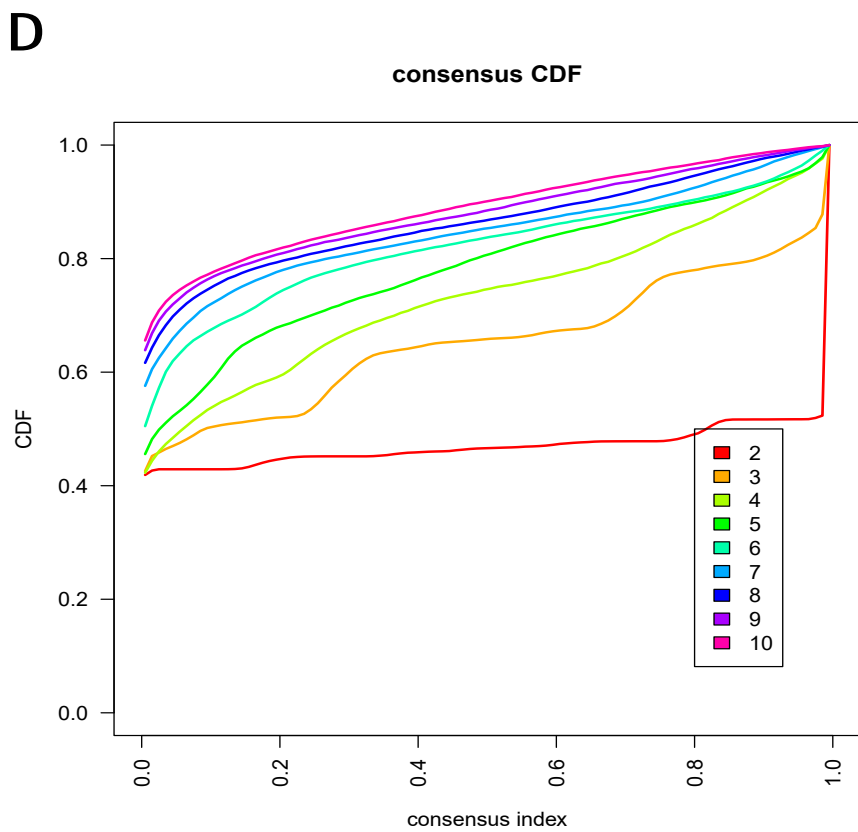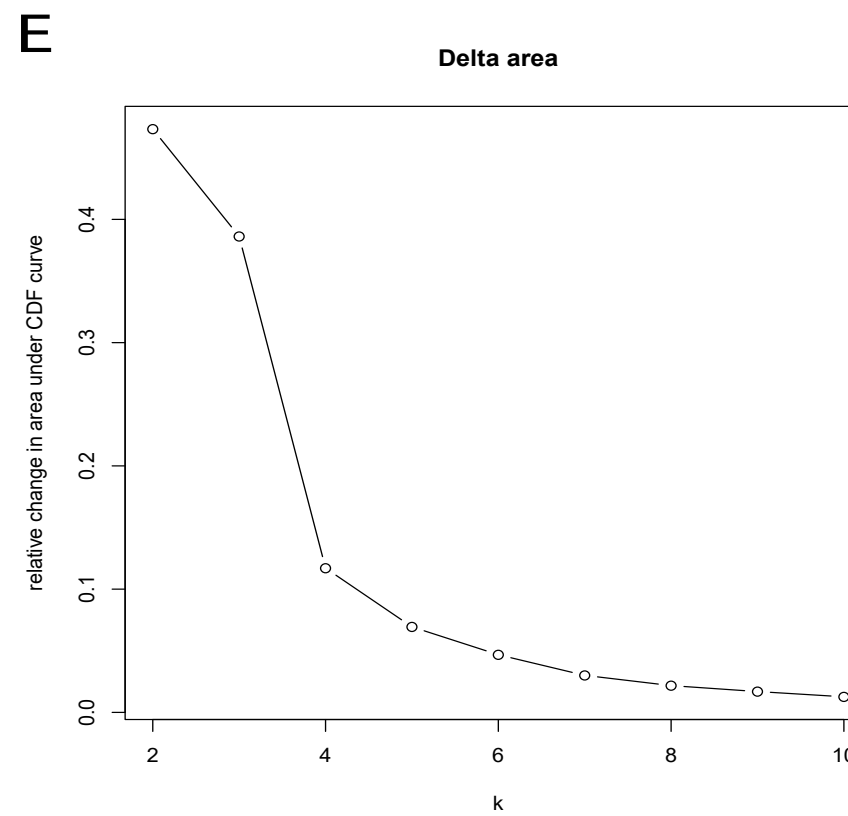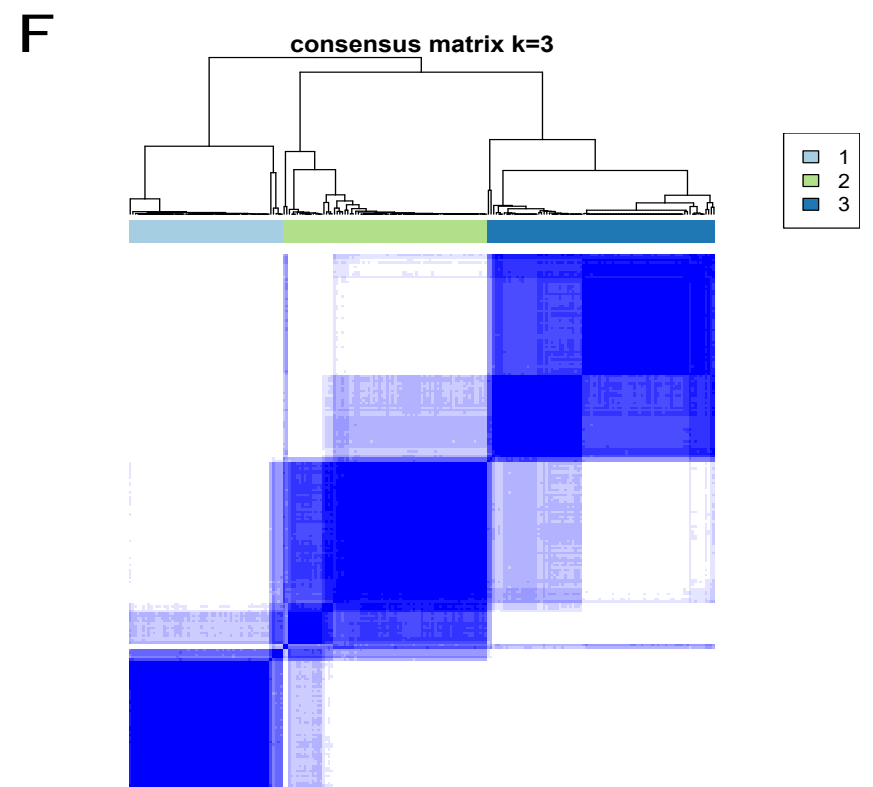

Supplement: Supplementary file 3 [file DataSheet1.PDF]
