# Supplementary material for: Molecular subtypes and scoring tools related to Foxo signaling pathway for assessing hepatocellular carcinoma prognosis and treatment responsiveness
Source: Front Pharmacol. 2023 Aug 24;14:1213506. doi: 10.3389/fphar.2023.1213506 (PMC10483071; doi:10.3389/fphar.2023.1213506)
Supplement: Supplementary file 5 [file DataSheet1.docx]

Supplementary Figure S1: Consistency clustering analysis.

A: Consistent cumulative distribution function at k=2-10 in TCGA-LIHC. B: Delta area at k=2-10 in TCGA-LIHC. C: Consistent clustering heat map of samples at k=3 in TCGA-LIHC. D: Consistent cumulative distribution function at k=2-10 in GSE14520. E: Delta area at k=2-10 in GSE14520. F: Consistent clustering heat map of samples at k=3 in GSE14520.

Supplementary Figure S2: Immune infiltration activity score of TME in TCGA-LIHC.

Supplementary Figure S3: Construction and validation of FPS.

A: Trajectory of independent variable changes in LASSO COX analysis. B: 10-fold cross-validation of lambda. C: K-M curves of patients in the high and low FPS groups in GSE14520. D: ROC curves for predicting 1-year, 3-year, and 5-year survival of HCC in GSE14520.
